# Assessing the Aerial Interconnectivity of Distant Reservoirs of *Sclerotinia sclerotiorum*

**DOI:** 10.3389/fmicb.2018.02257

**Published:** 2018-09-25

**Authors:** Christel Leyronas, Cindy E. Morris, Maria Choufany, Samuel Soubeyrand

**Affiliations:** ^1^Pathologie Végétale, INRA, Montfavet, France; ^2^BioSP, INRA, Avignon, France

**Keywords:** airborne inoculum, air-mass movement, contact network, polyphagous fungi, risk forecasting

## Abstract

Many phytopathogenic fungi are disseminated as spores via the atmosphere from short to long distances. The distance of dissemination determines the extent to which plant diseases can spread and novel genotypes of pathogens can invade new territories. Predictive tools including models that forecast the arrival of spores in areas where susceptible crops are grown can help to more efficiently manage crop health. However, such models are difficult to establish for fungi with broad host ranges because sources of inoculum cannot be readily identified. *Sclerotinia sclerotiorum*, the pandemic agent of white mold disease, can attack >400 plant species including economically important crops. Monitoring airborne inoculum of *S. sclerotiorum* in several French cropping areas has shown that viable ascospores are present in the air almost all the time, even when no susceptible crops are nearby. This raises the hypothesis of a distant origin of airborne inoculum. The objective of the present study was to determine the interconnectivity of reservoirs of *S. sclerotiorum* from distant regions based on networks of air mass movement. Viable airborne inoculum of *S. sclerotiorum* was collected in four distinct regions of France and 498 strains were genotyped with 16 specific microsatellite markers and compared among the regions. Air mass movements were inferred using the HYSPLIT model and archived meteorological data from the global data assimilation system (GDAS). The results show that up to 700 km could separate collection sites that shared the same haplotypes. There was low or no genetic differentiation between strains collected from the four sites. The rate of aerial connectivity between two sites varied according to the direction considered. The results also show that the aerial connectivity between sites is a better indicator of the probability of the incoming component (PIC) of inoculum at a given site from another one than is geographic distance. We identified the links between specific sites in the trajectories of air masses and we quantified the frequencies at which the directional links occurred as a proof-of-concept for an operational method to assess the arrival of airborne inoculum in a given area from distant origins.

## Introduction

Many phytopathogenic fungi are disseminated via the atmosphere from micro- to macro-geographical scales in the form of spores (Aylor et al., 1982; Brown and Hovmoller, 2002; Prospero et al., 2005). The most striking examples of long distance dispersal (LDD) can be illustrated by intercontinental movements of spores leading to rust emergence in previously unaffected areas. Evidence for atmospheric transoceanic jumps has been reported for coffee leaf rust, sugarcane rust and wheat stem rust, for example (Bowden et al., 1971; Purdy et al., 1985; Brown and Hovmoller, 2002). Long distance dispersal can also lead to the annual recolonization of some areas by periodic migrations of aerial spores. Indeed some pathogens make long distance jumps from one susceptible host to another throughout the growing season following prevailing winds. This is, for example, the case of Oomycetes responsible for tobacco blue mold and cucurbit downy mildew. It is also the case for cereal stem rust that migrates following the “*Puccinia* pathway" in the United States wheat belt, or for wheat yellow rust in China (Aylor et al., 1982; Hovmoller et al., 2002; Ojiambo and Holmes, 2011). Finally, LDD can lead to the invasion of areas where plant disease is already present by novel strains. This is the recent case for Ug99, a race of *P. graminis tritici*, which is virulent on cultivars of wheat carrying a particular, widely used resistance gene thereby making it an important threat to world wheat production. Modeled wind trajectories indicate that air movements are likely responsible for the spread of Ug99 in Africa and for its predicted trajectory into Asia (Singh et al., 2011; Meyer et al., 2017).

Forecasting the movements of fungal pathogens in the atmosphere is useful for anticipating epidemic risks. To forecast the arrival of spores in areas where susceptible crops are grown, local and distant sources of spores need to be identified and air-mass trajectories modeled (Isard et al., 2005; Tao et al., 2009). Predictive tools can help growers in rationalizing their practices, particularly chemical control, and thus can lead to a reduction in the number of fungicide applications. Studying atmospheric pathways frequently taken by plant pathogens may also be useful in organizing crops in regional landscapes, e.g., to reduce the cultivation of identical susceptible varieties in areas located on a dispersal route of a threatening pathogen (Singh et al., 2015; Meyer et al., 2017). Forecasting epidemic risks can be achieved for host-specific, obligate parasitic fungi such as rust or some oomycetes whose sources, and hosts along their trajectory can be readily identified (IPM-PIPE) (Isard et al., 2005; Tao et al., 2009). Indeed, each of these parasitic fungal species is able to grow on a very limited number of plant species. Moreover the fungi colonize only living host plants and cannot live saprophytically. Thus the identification of fields that are potential sources or sink of spores can be readily determined.

In contrast with host specific rusts and oomycetes, many phytopathogenic fungi are generalists with many environmental sources including host plants as well as substrates for saprophytic growth. Therefore, in such cases forecasting spore arrival is particularly difficult because sources of inoculum cannot be readily identified. Some fungal spores have been found up to several kilometers above ground level, above oceans or far away from any sources (Hirst et al., 1967; Maldonado-Ramirez et al., 2005; Prospero et al., 2005; Smith et al., 2012; Damialis et al., 2017) indicating long-distance dispersal events. Tracking the movements in the atmosphere of phytopathogenic fungi with large host ranges, in order to forecast epidemic risk, is challenging.

*Sclerotinia sclerotiorum*, the pandemic causal agent of white mold disease, can attack more than 400 plant species including some with economic importance such as field crops and vegetables (Purdy, 1979; Boland and Hall, 1994). Monitoring of airborne inoculum of *S. sclerotiorum* in several French cropping areas has shown that viable ascospores were present in the air almost all the time, even when there were no susceptible crops nearby suggesting that spores were from distal sources. This raises the hypothesis of a distant origin of airborne inoculum. Several approaches can be used to assess the pathway of spores over a large area (Golan and Pringle, 2017). Whereas monitoring fungal propagules with air samplers can give an idea about abundance of spores at a definite time in a definite location, it has limited application for assessing LDD. Molecular approaches allow comparative genetics of populations across a geographic range. However, they give little information about dispersal mechanisms and about the moment (recent or more ancient) when they occurred. Atmospheric transport models can be used for back-trajectory analysis to determine the origin of air masses and establish source–receptor relationships (Stein and Ngan, 2015a) while taking into account the errors of these estimates. Finally the most efficient methods to assess LDD of fungal spores are the ones that combine the three approaches (Golan and Pringle, 2017).

The objective of this study was to establish a method to determine the relationships of reservoirs of *S. sclerotiorum* and if certain strains are likely to be exchanged between regions located several 100 km apart. Based on air samplings, genetic characteristics of regional populations were compared. Aerial interconnectivity (networks of the air masses that would have transported spores aerially) was assessed by using archived meteorological data providing information about air mass movements.

## Materials and Methods

### Sampling of Viable Airborne Inoculum

Air sampling was carried out at four distinct regions of France between 2014 and 2016 (**Table 1**). Airborne propagules were collected using a volumetric sampler called Portable Air Sampler for Agar Plates (Burkard Manufacturing, Rickmansworth, United Kingdom) with a flow rate of 20 L min^−1^. Petri plates were filled with a semi-selective medium amended with bromophenol blue (Steadman et al., 1994). The samplers were set to run for 9 min twice during the day at each sampling date. One plate was used for each 9 min sampling. Samplers were placed in the North region of France (N) in a chicory witloof field (*Cichorium intybus* L.), in the Center-West region (CW) in a cantaloupe field (*Cucumis melo* L.) and in the South-West (SW) and North-West regions (NW) in carrot fields (*Daucus carota* L.).

**Table 1:** Genetic characteristics of *S. sclerotiorum* isolates collected in the air at four different French regions.

	Sample size	Year of collection	Nb of distinct MLH^a^	Haplotypic diversity	Allelic richness^b^	Hnb^c^
Total	498	2014–15–16	241	0.48	5.36	0.62 (0.19)
North	105	2014–15–16	59	0.55	4.47	0.59 (0.18)
North–West	18	2014–15–16	17	0.94	4.13	0.60 (0.17)
Center–West	49	2014–16	37	0.75	5.68	0.64 (0.21)
South–West	326	2014–15	154	0.47	5.27	0.60 (0.20)

*^a^Multilocus haplotype. ^b^Unbiased allelic richness corrected for a minimum sample size of 18 isolates. ^c^Unbiased gene diversity (standard deviation between brackets).*

After exposure, the plates were incubated in the lab at room temperature (ca 22°C) and the presence of *Sclerotinia* sp. on the selective medium was assessed by the development of yellow halos caused by the production of oxalic acid (Steadman et al., 1994). Mycelial colonies associated with yellow halos were transferred to fresh PDA (Potato Dextrose Agar) to obtain pure cultures. Isolates showing the typical morphological features of *Sclerotinia* sp. were subjected to a step of single hypha isolation prior to their entry in the fungal collection of the laboratory after which they were considered as strains (i.e., pure lines). For this, single pieces of hyphal tip were excised from the growing margin of a colony after 2 days of incubation on PDA and transferred to fresh medium as described by Lehner et al. (2016). The single-hypha strains were then stored as sclerotia at −20°C.

### Genotyping

Sclerotia, typical mycelial stroma formed by *S. sclerotiorum*, were spread on PDA to germinate and produce mycelium. Genomic DNA was extracted in 96-well plates from aliquots of 100 mg (fresh weight) of frozen mycelium, following the Dneasy Plant extraction Kit protocol (Qiagen). Sixteen microsatellite markers designed for *S. sclerotiorum* by Sirjusingh and Kohn (2001) were amplified with forward primers conjugated with fluorescent dyes following the protocol described by Leyronas et al. (2018). Reverse primers did not carry any fluorescent. To determine the size of the microsatellites, the PCR products were diluted and multiplexed prior to scanning with an ABI 3730 sequencer (Applied Biosystems). GeneMapper software version 4.1 (Applied Biosystems) was then used for the microsatellite size analysis. Complete microsatellite size profiles (referred to as “haplotypes” hereafter) were obtained for all strains.

### Genetic Characterization of Airborne *S. sclerotiorum* Strains

Unbiased gene diversity (Hnb) and unbiased allelic richness were computed separately for the strains collected in distinct regions with the Genetix software (Belkhir et al., 1996–2004). The number of different multilocus haplotypes (MLH) was computed with GenClone 1.0 software (Arnaud-Haond and Belkhir, 2007). We used the index of haplotypic diversity (based on the number of individuals and the number of distinct MLH), which estimates the proportion of haplotypes present in a population and is equal to 1 when a population is composed exclusively of unique haplotypes (Arnaud-Haond et al., 2007). The software FSTAT version 2.9.3 (Goudet, 1995) was used to compute allelic richness per locus corrected for a minimum sample size of 18 isolates. Genetic differentiation was estimated with a hierarchical analysis of molecular variance (AMOVA) using Arlequin version 3.5 (Excoffier et al., 2005). AMOVA was used to determine the proportion of genetic variation partitioned among the four regions, among the years of collection within a region, or among isolates within a sampling year. The number of permutations to test for significance was set at 8,000. Arlequin was also used to assess genetic differentiation between strains of pairs of regions by computing *R*_ST_ values (Slatkin, 1995).

### Two-Part Decomposition of an Observed Pathogen Composition

**FIGURE 1:**
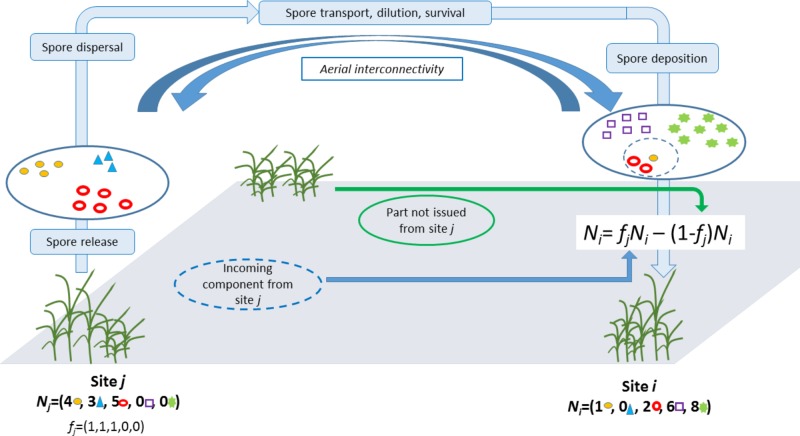
Aerobiological phases of spore dispersal and composition of aerial samples.

To decompose the composition of aerial samples into strains of local and distant origins we used the following logic:

We let *N_i_* = (*N*_*i*1_,...,*N_in_*) denote the counts of isolates from strains 1, ..., *n* collected from site *i*. Then *N_i_* was split into two parts: the part issued from site *j* (via long-distance dispersal) and a part not issued from site *j* (i.e., either generated locally or issued from other distant sites) (**Figure 1**). Here, we make the crude assumption that for any strain *s* that was collected in both sites *i* and *j*, the corresponding count *N_is_* is issued from site *j* and, reversely, for any strain *s* that was collected in site *i* but not in site *j*, the corresponding count *j* is not issued from site *j*. Mathematically, this translates into: *N_i_* = *f_j_N_i_* − (1 − *f_j_*) *N_i_* where *f_i_* is a vector of dimension *n* whose component *s* is equal to 1 if strain *s* was collected in site *j*; otherwise it equals 0. Thus, *f_i_* plays the role of a filter and *f_i_N_i_* stands for the part in *N_i_* issued from site *j* whereas (1 − *f_j_*)*N_i_* stands for the part in *N_i_* not issued from site *j*. The specification used here for *f_i_* implies that all strains in *j* have an equal propensity to be dispersed from *j* to *i* and an equal propensity to establish in *i*.

How likely *f_j_N_i_* issues from site *j* can be measured by assuming that this vector of counts is drawn from a multinomial distribution with the vector of probability*p_j_* = *N_j_*/$\sum_{s=1}^{n}N_{\mathrm{js}}$, i.e., the proportion of the strain in site *j*. We let *q*_*j*→*i*_ denote the multinomial probability computed at *f_j_N_i_* with parameter *p_j_*. Thereafter, *q*_*j*→*i*_ is called the probability of the incoming component (PIC) in site *i* from site*j*.

To avoid inconsistencies because of varying sample sizes at different sites, probability *q*_*j*→*i*_ was computed by subsampling with replacement *k* isolates in *N_i_* and *N_j_*, where *k* is the minimum sample size across all sites (not only *i* and *j*), computing $f_{j}^{*}$ and $p_{j}^{*}$ from the subsampled vectors of counts, say $N_{i}^{*}$ and $N_{j}^{*}$, and computing the PIC $q_{j\to i}^{*}$ in site *i* from site *j* using $N_{j}^{*}$ and a subsample of size 1 of $N_{i}^{*}$. This procedure was repeated 10^4^ times and the average of the 10^4^ $q_{j\to i}^{*}$ runs was used as the value of *q*_*j*→*i*_.

### Relationship Between Incoming Components of the Pathogen Population and Tropospheric Connectivity

We aim to explore the association between (a) the probability that the different components of the population sampled from the air are incoming from elsewhere (PIC) and (b) the connectivity between sites via tropospheric movements. The rationale is that the greater the connectivity, the greater the PIC. This association was assessed with a log-linear regression:

*logq*_*j*→*i*_ = α + β*C*_*j*→*i*_ + ε_*j*→*i*_ where *C*_*j*→*i*_ measures the directional connectivity from site *j* to site *i*, α is the intercept coefficient, β is the slope coefficient and ε_*j*→*i*_ is random noise.

Directional connectivity was inferred by exploiting archived meteorological data provided by the Global Data Assimilation System (GDAS) of NOAA and the software HYSPLIT (Stein et al., 2015b; Rolph et al., 2017) that allows the modeling of air mass trajectories from the aforementioned data. For each date between 2008-01-01 and 2017-12-31, i.e., a 10 years period over which GDAS data are available, HYSPLIT allowed us to reconstruct over 48 h the trajectory *H*(*t,x*) of the air mass arriving at location × (1,000 m above the sea) and date t (12:00 GMT). We computed the connectivity from site *j* to site *i* as the fraction of trajectories arriving at site *i* and previously going throughout a buffer zone around site *j*. Here, the connectivity is considered in a multisite context, and can therefore be expressed in the graph theory framework. A graph *G* = (*V,E*) is characterized by a set *V* of vertices and a set *E* of links between vertices, i.e., the edges of the graph. In our application, the vertices correspond to the sampling sites and the edges are weighted by the possibility of pathogen diffusion between two different sites via air mass movements, namely the directional connectivity terms. For our application, we calculated different connectivities corresponding to the 12 months of the year. Thus, each monthly connectivity value was based on approximately 300 (10 years × 30 days) binary variables indicating whether or not an air mass arriving at a given site went through the vicinity of another given site. More precisely, the monthly connectivity for the *m*-th month of the year from site *j* to site *i* was computed as follows:

$$C_{j\to i}^{\left(m\right)}=\frac{1}{K_{m}}\sum_{k=1}^{K_{m}}1(H(x_{i},t_{\mathrm{mk}})\cap B(x_{j},r)\neq \emptyset$$

where {*t_mk_* : *k* = 1,...,*K_m_*} are all dates of the *m*-th month of the year between 2008-01-01 and 2017-12-31, *B* (*x_j_*, *r*) is a circular buffer of radius *r* around site *j*, and 1 (*H* (*x_i_*, *t_mk_*) ∩ *B* (*x_j_*, *r*)) is the indicator function equal to 1 if the intersection between the trajectory *H* (*x_i_*, *t_mk_*) and the buffer *B* (*x_j_*, *r*) is not empty; otherwise it equals to 0. In our application, buffer zones were defined as disks of radius 25 km centered on sampling sites, and allowed us to take into account the uncertainty in the reconstruction of air mass trajectories. The use of buffer zones also implies that an assumption of local stability in pathogen composition is made. Thereafter, for the sake of simplicity in the notation, we omit the index m.

The log-linear regression between *q*_*j*→*i*_ and *C*_*j*→*i*_ was fitted to data with a least-squares approach. To achieve a satisfactory statistical power, for each pair (*i,j*) we only used the point (*q*_*j*→*i*_, *C*_*j*→*i*_) or (*q*_*j*→*i*_, *C*_*j*→*i*_) corresponding to the maximum connectivity. The significance of the link was assessed via a randomization directional test (Manly, 1997) assessing whether the slope coefficient β is equal to 0, the alternative being that β is positive. Under randomization, the regression is expected to be flat (i.e., β = 0), with some variations. For each test, 10^4^ randomizations were independently carried out by conserving the network structure of data: for each repetition, identifiers of sites were simply randomly re-sampled without replacement for the connectivities and the following log-linear regression was fitted to the randomized dataset:

*logq*_*j*→*i*_ = α + β$C_{j\to i}^{*}$ + $\varepsilon_{j\to i}^{*}$ where $C_{j\to i}^{*}$ denotes the randomized directional connectivity from site *j* to site *i*. The *p*-value of the test was computed as the proportion of estimates β^^∗^^ obtained for the 10^4^ repetitions larger than the estimate β obtained with non-randomized data.

### Simulation Study

A simulation study was designed to assess the efficiency of the method aimed at linking the tropospheric connectivity with the PIC and to compare different sampling schemes varying in the number of sampling sites, *I*, and the number of sampled isolates per site, *M*. For this goal, we generated datasets from an empirical simulation model that does not represent real processes, but produces multi-site pathogen compositions with various levels of similarities depending on arbitrarily generated connectivities.

In the model, for any target site i and any source site *j* ≠ *i*, the tropospheric connectivity *C*_*j*→*i*_ was drawn in an exponential distribution with mean 0.02. Thus, 95% of connectivities are lower than 0.060, and 99% of connectivities are lower than 0.092.

Each site *i* was supposed to be occupied by a proportion 1 − τ_*i*_ of endemic strains of the pathogen and a proportion τ_*i*_ of exogenous strains, where τ_*i*_ depends on the connectivities toward site *i* and a parameter, τ_*max*_, giving the maximum exogenous fraction of strains of the pathogen at the site. The set of endemic strains in *i* consists of *K* strains with random proportions. The set of exogenous strains in *i* is potentially made of all the strains that are endogenous in distant sites *j* ∈ {1,...,*I*} − {*i*} such that *C*_*j*→*i*_ > 0 (here, for the sake of simplicity, we suppose that the set of I sampling sites form a closed system).

More precisely, pathogen compositions *N_i_* in site *i* ∈ {1,...,*I*} were drawn with the following three-stage procedure.

(1) The proportion τ_*i*_ of exogenous strains satisfies.
(2) τ_*i*_ = τ_*max*_$\frac{C_{\cdot \to i}}{\sum_{i^{\prime }=1}^{I}C_{\cdot \to i^{\prime }}}$,
(3) where *C*_⋅→*i*_ = ∑_*j*≠*i*_*C*_*j*→*i*_ denotes the overall connectivity of site *i*.
(4) Proportions γ_(*i*−1)*K*+1_,…,γ_(*i*−1)*K*+*K*_ of the *K* endemic strains in site *i*, that are indexed by (*i*−1)*K* + 1,…,(*i*−1)*K* + *K*, are randomly generated by simulating *K* independent values *U_ik_* (*k* = 1,…,*K*) in the uniform distribution over [0,1] and setting γ_(*i*−1)*K*+*k*_ = *U_ik_*/$\sum_{k^{\prime }=1}^{K}U_{ik^{\prime }}$. Then, counts of sampled isolates of endemic strains in site *i*, denoted by *N*_*i*,(*i*−1)*K*+1_,…,*N*_*i*,(*i*−1)*K*+*K*_, are obtained by a multinomial draw with size [(1 − τ_*i*_) *M*] and probabilitiesγ_(*i*−1)*K*+1_,…,γ_(*i*−1)*K*+*K*_, where the rounding operator [•] is applied to obtain integer values.
(5) Finally, counts of sampled isolates of exogenous strains in site *i* are obtained by a multinomial draw with size [τ_*i*_*M*] and vector of probabilities proportional to Δ, whose element (*j* − 1) *K* + *k*, *k* = 1,…,*K*, is equal to zero if *j* = *i*, and equal to γ_(*j*−1)*K*+*k*_*C*_*j*→*i*_ otherwise:
(6) $\Delta_{(j-1)K+k}=\{\begin{array}{ccc} 0 & \mathrm{if} & j=i\\ \gamma_{(j-1)K+k}C_{j\to i} & \mathrm{if} & j\neq i \end{array}$

Hence, the probability that an endemic strain of *j* is sampled in *i* depends on the relative importance of this strain in *j* and the connectivity from *j* to *i*.

Using the empirical simulation model described above, we made several series of 100 simulations with varying values of the number of sites *I*, the number of isolates per site *M* and the maximum exogenous fraction of pathogen strains at the site level τ_*max*_. Then, we carried out two comparisons:

(1) We compared the situation where (*I*, *M*) = (4,250) (i.e., a few sites with large samples) and the situation where (*I*, *M*) = (10,100) (i.e., a larger number of sites with smaller samples); 1,000 isolates were sampled in both situations. For this comparison we set τ_*max*_ = 0.2 (i.e., the maximum proportion of exogenous strains at the site level is 20%) and *K* = 10 (i.e., each site has 10 endemic strains).
(2) Then, we studied the case where the maximum proportion of exogenous strains at the site level is low, namely 2% (τ_*max*_ = 0.02) and compared two different sampling efforts: (*I*, *M*) = (10,100) and (*I*, *M*) = (10,1000). For this comparison, *K* = 10.

## Results

### Genetic Characteristics of Airborne *S. sclerotiorum* Strains

**FIGURE 2:**
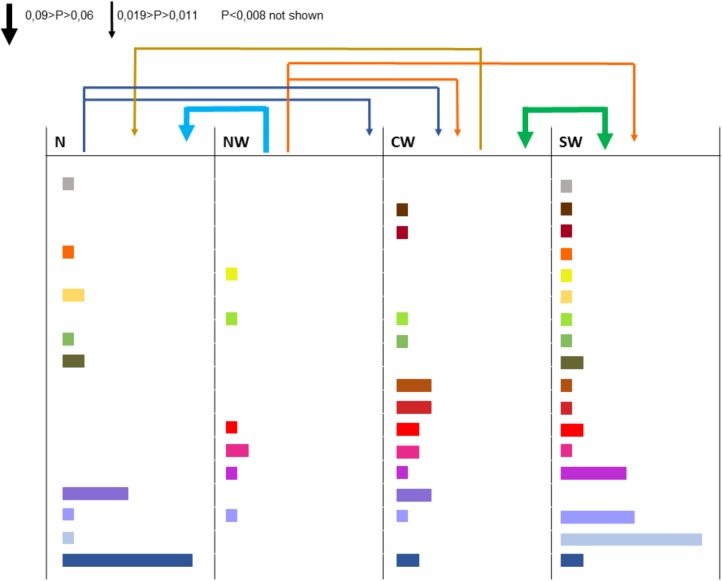
Strains collected from different regions and haplotypes they have in common (each color corresponds to one haplotype, the width of the rectangle indicates the number of strains bearing this haplotype). The arrows above columns indicate the aerial connectivity between regions (the larger the arrow, the more intense the connection).

All the 16 markers used to characterize the 498 strains were polymorphic with a number of alleles ranging from 2 (for locus 36-4) to 21 (for locus 106-4). Strains collected from the Center-West region showed the highest gene diversity and allelic richness (**Table 1**). A total of 241 different multilocus haplotypes (MLH) were identified, of which 172 were represented by only one strain. Our analyses of dissemination and connectivity were based on the 69 MLH that were represented by at least 2 strains. The most frequent MLH was represented by 20 strains, all coming from the South-West. Furthermore, 18 of the MLH represented by multiple strains were collected from more than one region (**Figure 2**). The geographic distance between the collection sites of strains sharing the same haplotype was as far as 700 km.

Hierarchical AMOVA revealed that 89.2% of the genetic variability was distributed within sampling years, compared to 1.6% within a region and 9.2% among regions. These three factors contributed significantly to total genetic variance (*P* < 0.05).

There was no evidence of genetic differentiation between *S. sclerotiorum* strains with the *R*_ST_ fixation index statistic not significant (*P* > 0.05) for pairwise combination SW-CW, SW-NW, CW-NW and NW-N (clonally-corrected data). There was evidence of low genetic differentiation between *S. sclerotiorum* strains with the *R*_ST_ highly significant (*P* < 0.01) for pairwise combination North/Center-West (*R*_ST_ = 0.094 and 0.064 with clonally corrected data) and North/South-West (*R*_ST_ = 0.152 and 0.099 with clonally corrected data). There was also a low genetic differentiation (value < 0.10) between *S. sclerotiorum* strains from the North and the North-West (*R*_ST_ = 0.061, *P* = 0.03) for the non-corrected dataset.

### Tropospheric Connectivity

**FIGURE 3:**
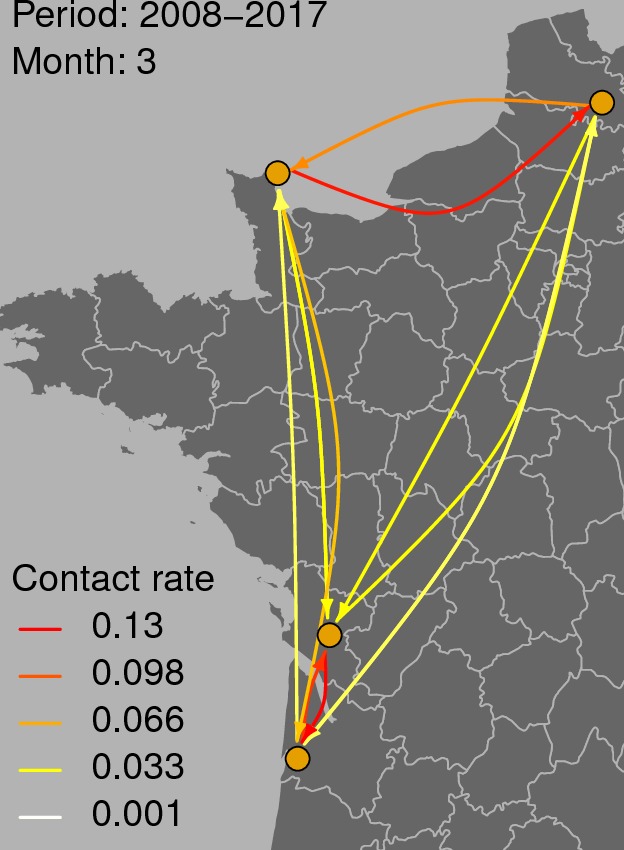
Tropospheric connectivity in March that was inferred with HYSPLIT from archived meteorological data over the period 2008–2017.

**FIGURE 4:**
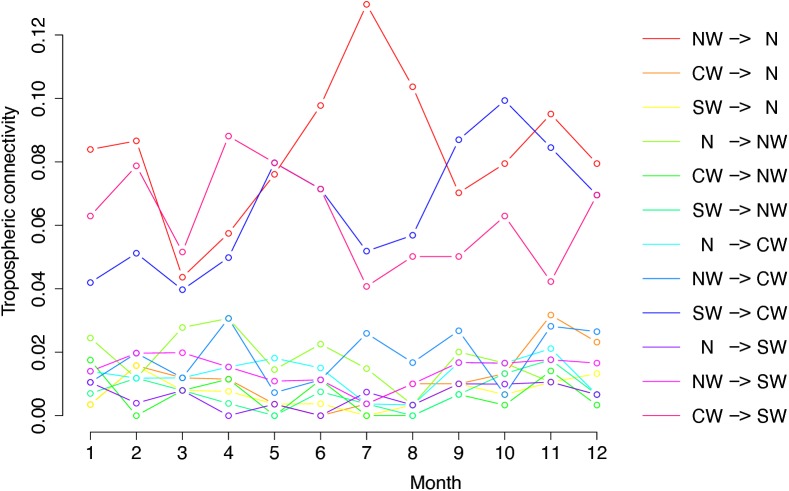
Temporal variation in the tropospheric connectivity between sites.

**FIGURE 5:**
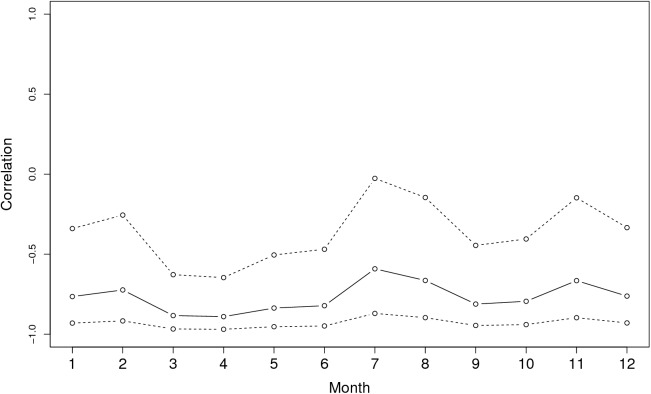
Correlation across time between the tropospheric connectivity and the geographic distance.

**Figure 3** shows the graph of tropospheric connectivity in March that was inferred with HYSPLIT from archived meteorological data over the period 2008–2017. Our approach provides an assessment of the directional links between the sites where *S. sclerotiorum* has been sampled. Thus, we obtained, for each month of the year, a network with 4 nodes and 12 edges with varying intensities (**Supplementary Figures S1–S11** are graphs for months other than March). Directionality in the links can be clearly seen on these graphs. **Figure 4** gives a clearer assessment of the variation in the connectivity with time. Despite temporal variation in the directional connectivity between two given sites, we observe stability with respect to the most connected sites (three pairs of sites with directional connectivity larger than 0.04) and the less connected sites (nine pairs of sites with directional connectivity lower than 0.03). The maximum connectivity is about 0.13 between NW and N sites in July (i.e., 13% of air mass trajectories arriving in the N site in July go through the buffer zone around site NW). The connectivity NW→N is the largest for 7 months of the year but the connectivity SW→CW and CW→SW are also occasionally the highest ones. The correlation across time between the tropospheric connectivity and the Earth surface distance is displayed in **Figure 5**. This correlation around -0.75 indicates that the two variables are connected but additional information is included in the tropospheric connectivity, in particular the directionality.

### Link Between PIC and Tropospheric Connectivity

**FIGURE 6:**
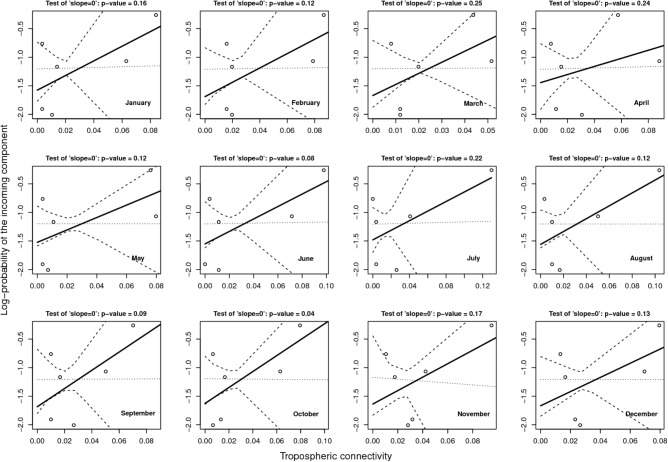
Log-linear regression between PIC and tropospheric connectivity.

**FIGURE 7:**
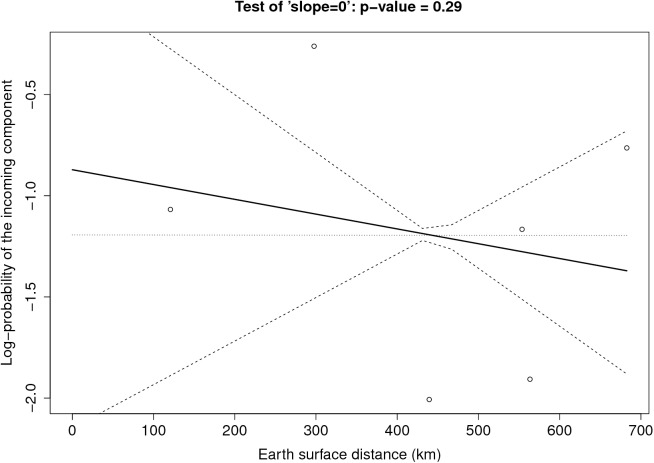
Log-linear regression between the PIC and the Earth surface distance.

**Figure 6** provides the result of the log-linear regression between the PIC and the tropospheric connectivity. The regression lines are rather stable across time, reflecting the relative stability of the tropospheric connectivity. They indicate a positive trend in the link between the PIC and tropospheric connectivity, the test of “slope = 0” leading to low *p*-values for June, September, and October. However, the small number of sites does not allow us to definitely state that this link is clearly founded. The regressions could be influenced by extreme points. As a benchmark, we performed the same analysis between the PIC and the Earth surface distance (**Figure 7**), which are expected to be negatively correlated. The limitation due to the number of sites also applies here, but the link seems weaker in **Figure 7** than in **Figure 6**, suggesting that tropospheric connectivity is more appropriate to explain the probability of the incoming microbial component.

### Simulation-Based Assessment of Different Sampling Schemes

**FIGURE 8:**
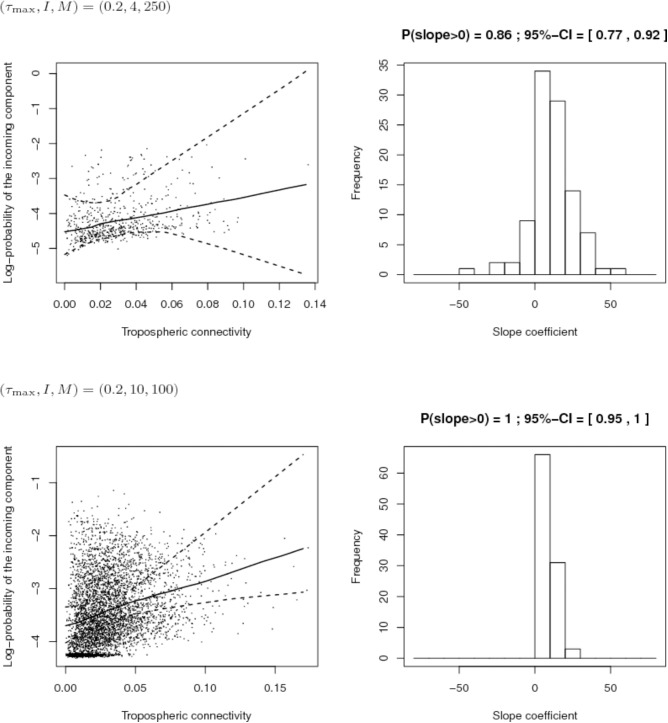
Comparison of the inferred links between connectivity and probability of the incoming component (PIC) for different sampling schemes (top: 4 sites and 250 isolates per site; bottom: 10 sites and 100 isolates per site) when the maximum proportion of exogenous strains at the site level is 20%. Left: Cloud of points “connectivity × PIC” obtained for the 100 simulations and pointwise median and quantiles of order 0.025 and 0.975 of the linear regression line linking connectivity and PIC. Right: histogram of the estimate of the slope coefficient in the regression (a positive slope tends to show a positive link between connectivity and PIC). The proportion of positive estimated slopes and its 95%-confidence interval are given above the histogram.

First, we compared the sampling schemes characterized by (*I*, *M*) = (4,250) and (*I*, *M*) = (10,100), when the maximum proportion of exogenous strains at the site level is rather high, i.e., τ_*max*_ = 0.2. **Figure 8** shows that the sampling scheme with more sites but less isolates per site leads more systematically to a positive estimate of the slope coefficient in the regression between the connectivity and the log-PIC. Using 10 sites instead of 4 allows, for each repetition, to have 10 × 9/2 = 45 points instead of 4 × 3/2 = 6 points to fit the regression. Obviously, there is a trade-off between the number of sites and the number of samples per site because a weak number of samples per site could lead to inaccurate estimates of PIC values and, therefore, inaccurate estimates of the slope. In addition, from a computational point of view, calculation time increases with the square of the number of sites.

**FIGURE 9:**
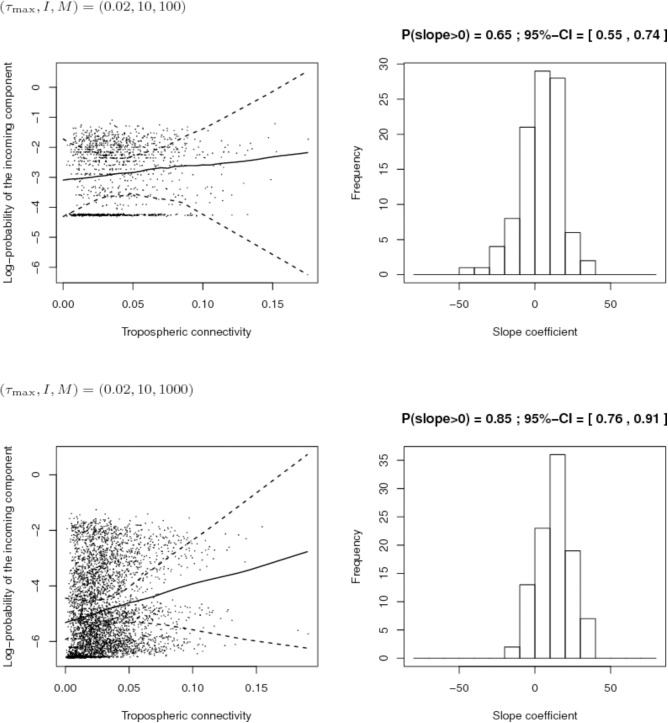
Comparison of the inferred links between connectivity and probability of the incoming component (PIC) for different sampling efforts (top: 10 sites and 100 isolates per site; bottom: 10 sites and 1,000 isolates per site) when the maximum proportion of exogenous strains at the site level is 2%. Left: Cloud of points “connectivity × PIC” obtained for the 100 simulations and pointwise median and quantiles of order 0.025 and 0.975 of the linear regression line linking connectivity and PIC. Right: histogram of the estimate of the slope coefficient in the regression (a positive slope tends to show a positive link between connectivity and PIC). The proportion of positive estimated slopes and its 95%-confidence interval are given above the histogram.

Second, we compared the sampling schemes characterized by (*I*, *M*) = (10,100) and (*I*, *M*) = (10,1000), when the maximum proportion of exogenous strains at the site level is rather low, i.e., τ_*max*_ = 0.2. **Figure 9** shows that using (*I*, *M*) = (10,100) with a 2% maximum proportion of exogenous strains at the site level is not enough to infer the link between connectivity and PIC. However, the power of the analysis tool significantly increases with the number of sampled isolates per site.

## Discussion

Variants of pathogens that migrate from one place to another can reduce the efficiency of strategies to manage plant health if the incoming variants are resistant to or less sensitive to the methods used at the site where they arrive. Furthermore, identification of the origin of incoming inoculum could help in predicting the arrival of inoculum and in reducing, where possible, the quantity of inoculum available from distal sources. For pathogens with a wide range of possible sources, the incoming component of inoculum cannot be monitored in real time in a given site. Therefore, we looked for indicators that were significantly linked to the probability of incoming airborne inoculum. In particular we developed a method to evaluate connectivity between distant sites via the atmosphere and assessed the relationship between aerial connectivity and incoming inoculum. This was based on data for daily air mass trajectories from which the airborne connectivity between sites and the prevailing directions of connectivity were inferred.

The results show that the rate of aerial connectivity between two sites varies according to the direction and to the month considered. For example, it was shown that during the period from 2008 to 2017, North and North–West regions were significantly connected but more strongly in the North-West toward North direction than the inverse. On the contrary, South–West and Center–West had high rates of connectivity but in both directions. Finally some regions had low rates of aerial connectivity (e.g., South–West toward North and North-West). This directional connectivity corresponds to air mass movements that change with the seasons – a notion that is obvious to anyone who is attentive to the weather as seasons change. The originality of this work is that we identified the links between specific sites that were in the trajectories of the air masses and we quantified the rates at which the different directions of the links occurred.

The results also show that aerial connectivity is more informative than geographic distance between two sites to explain the PIC of inoculum at a given site from another one. Aerial connectivity adds information about the direction of potential exchanges via the atmosphere that geographic distance cannot provide. This information is particularly interesting since it makes it possible to identify more precisely the role of each site as a source or sink of spores. It can therefore be hypothesized that isolates with identical haplotypes in two regions are more likely to have been emitted by the one having aerial connectivity directed toward the other (such as NW toward N for example).

The fact that aerial connectivity is more informative than geographic distance may be due to the movements of air masses between two sites that are not simply linked to linear distance they travel but also to topography, climate and the global movement of air masses on the terrestrial surface. Taking the example of NW and N regions of the present study: aerial connectivity reflects the general movements of air masses that often enter the French territories by the Atlantic coast and that move inland. In contrast, geographic distance (about 300 km) doesn’t give any information about frequency and direction of aerial exchanges between NW and N. In order for connectivity to be as informative as possible, we calculated it over several years to provide a global and robust vision of the prevailing links between two sites.

Studying and predicting airborne routes of plant pathogens is of general interest because atmospheric highways have very few boundaries. Recently, key airborne dispersal routes of rust between several African and Asian countries were studied by Meyer et al. (2017) using a mechanistic modeling framework. In their study, data about heterogeneous landscapes of wheat fields, turbulent atmospheric spore dispersal and environmental suitability for infection after deposition were computed. This model is well adapted to a host specific pathogen like rust but not for broad-host range fungi because sources cannot be easily identified. The method developed in the present study bypasses this pitfall by allowing us to assess aerial interconnectivity between sites that do not necessarily harbor crops.

The present study describes a method in its preliminary state. We used simulations to investigate how to improve this method and in particular how to optimize the balance between the number of sites considered and the number of strains collected at each site. These simulations revealed that to determine links between sites it is better to have more sites with few strains than few sites with many strains. However, a balance has to be found because an insufficient number of strains per site could lead to an inaccurate estimates of PIC values. This balance has to be considered according to the practical aspects of sampling (number of samplers to deploy, number of isolates to isolate and to genotype…). These simulations could be performed to establish the experimental design for future studies while taking into account the number of potential sampling sites and the effort needed to isolate and characterize strains of the targeted pathogen.

Moreover, the way the PIC was computed could be tuned to reflect more realistic processes. In this study, the PIC was obtained by introducing a filter *f_i_* that implies that all strains in *j* have an equal propensity to be dispersed from *j* to *i* and an equal propensity to establish in *i*. To weaken this assumption, we tested an alternative specification for the filter that incorporates the possibility for some strains of *j* to not be dispersed or not be adapted to site *i*: the filter, denoted by *f*_*j*→*i*_, is a vector of dimension *n* whose component *s* is equal to 1 if strain *s* was collected both in site *j* and *i*; otherwise it is 0. With such a specification, we, however, assume that all the strains in *j* that can be dispersed to and establish in *i* have an equal propensity to make it to *i*. The use of this alternative filter led to different PIC values in the real-case study tackled in this article, but did not yield qualitatively different results. Further investigation should be carried out to consider more relevant filters and obtain more accurate inferences about the long-distance dispersal of pathogens.

## Conclusion

Knowledge about connectivity (its rate and its direction between sites) at a regional and global scale is useful for establishing strategies to set up networks of epidemio-surveillance for wind-dispersed fungi. Practically, spore monitoring devices could be placed at existing sites of meteorological networks, for example, or on elevated buildings (i.e., lighthouses for example), or at stations for measurement of flux of atmospheric particles^1^. Placing trapping devices upstream of the North–West site described in this study, for example, along the French oceanic border could help to identify spores coming from outside the French territory. Information about airborne inoculum potentially arriving in a territory is important to obtain since the traits of exogenous strains may differ from endogenous ones and sometimes may lead to more damaging epidemics. For example, countries where the inoculum originated could have policies concerning the use of fungicides that are different from French policies. In this way, incoming fungal strains may have developed resistance to fungicides that have not yet been deployed in France or that are reserved for use in cases of extreme potential for crop damage. If such strains manage to infect and multiply in France, they may lead to the modification of the endogenous fungal population thereby rendering obsolete the use of certain fungicides. Different cropping systems or climates may also result in more aggressive or more robust strains than endogenous ones. Finally if an incoming fungal population of a given species is more diverse than the local endogenous population, the choice of cultivars might need to be modified otherwise resistance may be overcome by the first arrival of exogenous airborne inoculum. The tools and analytical methods that we have presented here can be further developed to address the impact of aerial inoculum from long distance sources and the capacity to account for this in strategies of crop protection.

## Author Contributions

CL carried out microsatellite genotyping and genetic data analysis. CM and SS developed the initial idea of tropospheric interconnectivity. SS and MC developed and conducted the calculations concerning tropospheric interconnectivity and pathogen composition decomposition. CL coordinated the writing of the manuscript. CL, CM, MC, and SS wrote the manuscript. All authors reviewed the manuscript.

## Conflict of Interest Statement

The authors declare that the research was conducted in the absence of any commercial or financial relationships that could be construed as a potential conflict of interest.

## Footnotes

1 https://daac.ornl.gov/FLUXNET/guides/Fluxnet_site_DB.html
