# Supplementary figures and images for: Assessing the Aerial Interconnectivity of Distant Reservoirs of Sclerotinia sclerotiorum
Source: Front Microbiol. 2018 Sep 25;9:2257. doi: 10.3389/fmicb.2018.02257 (PMC6178138; doi:10.3389/fmicb.2018.02257)

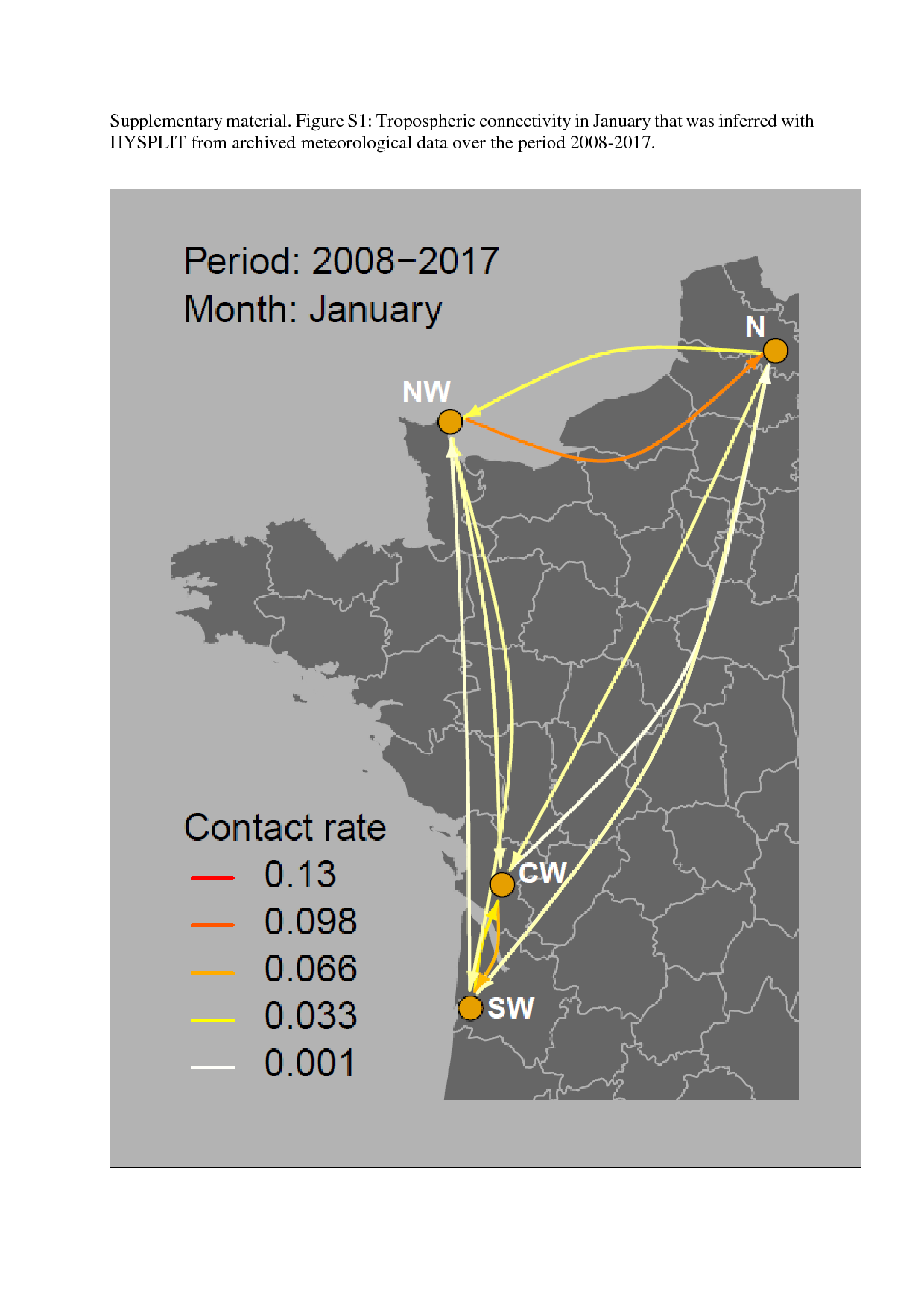

Supplement: Supplementary file 1 [file Image_1.TIFF]

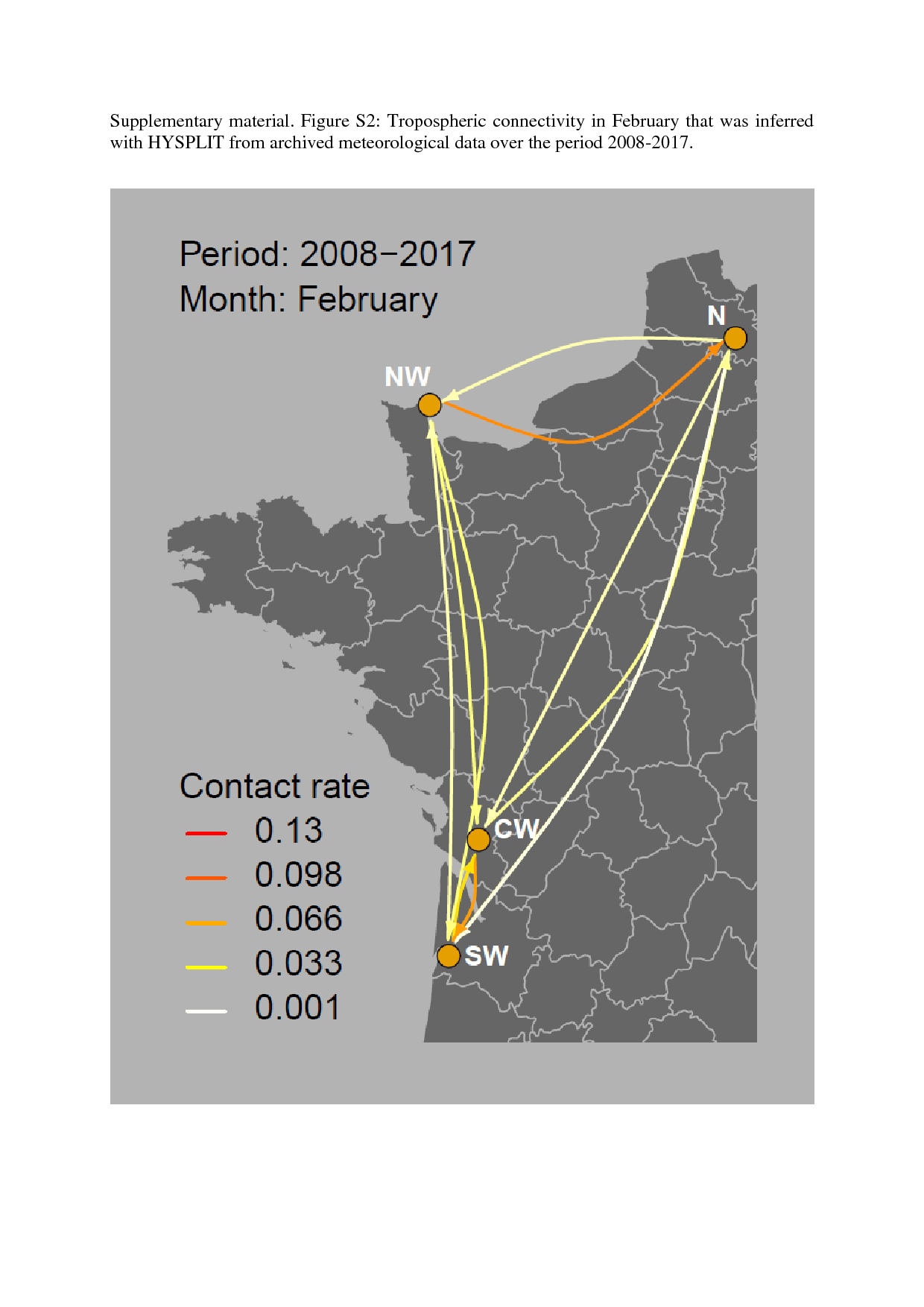

Supplement: Supplementary file 2 [file Image_2.TIFF]

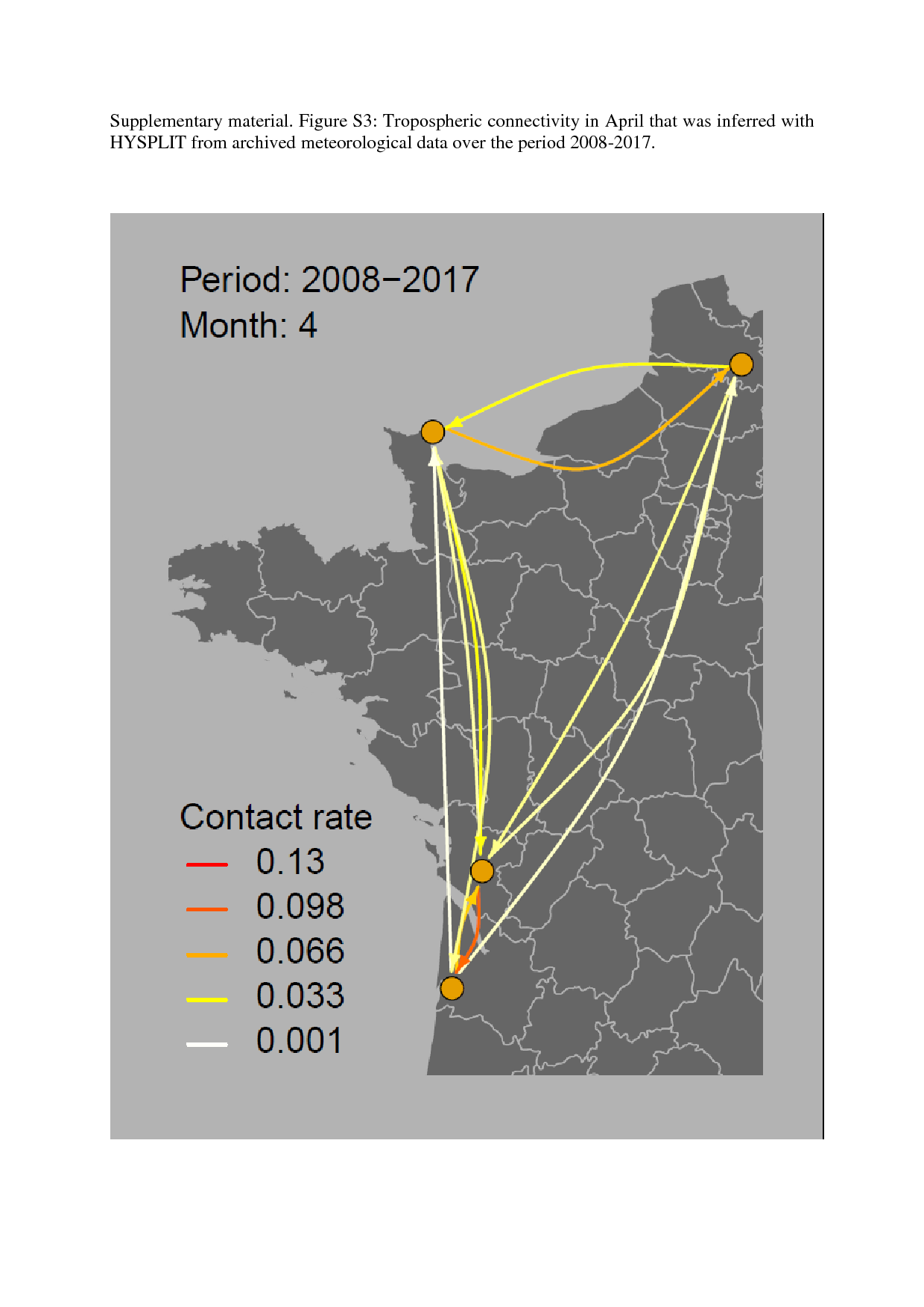

Supplement: Supplementary file 3 [file Image_3.TIFF]

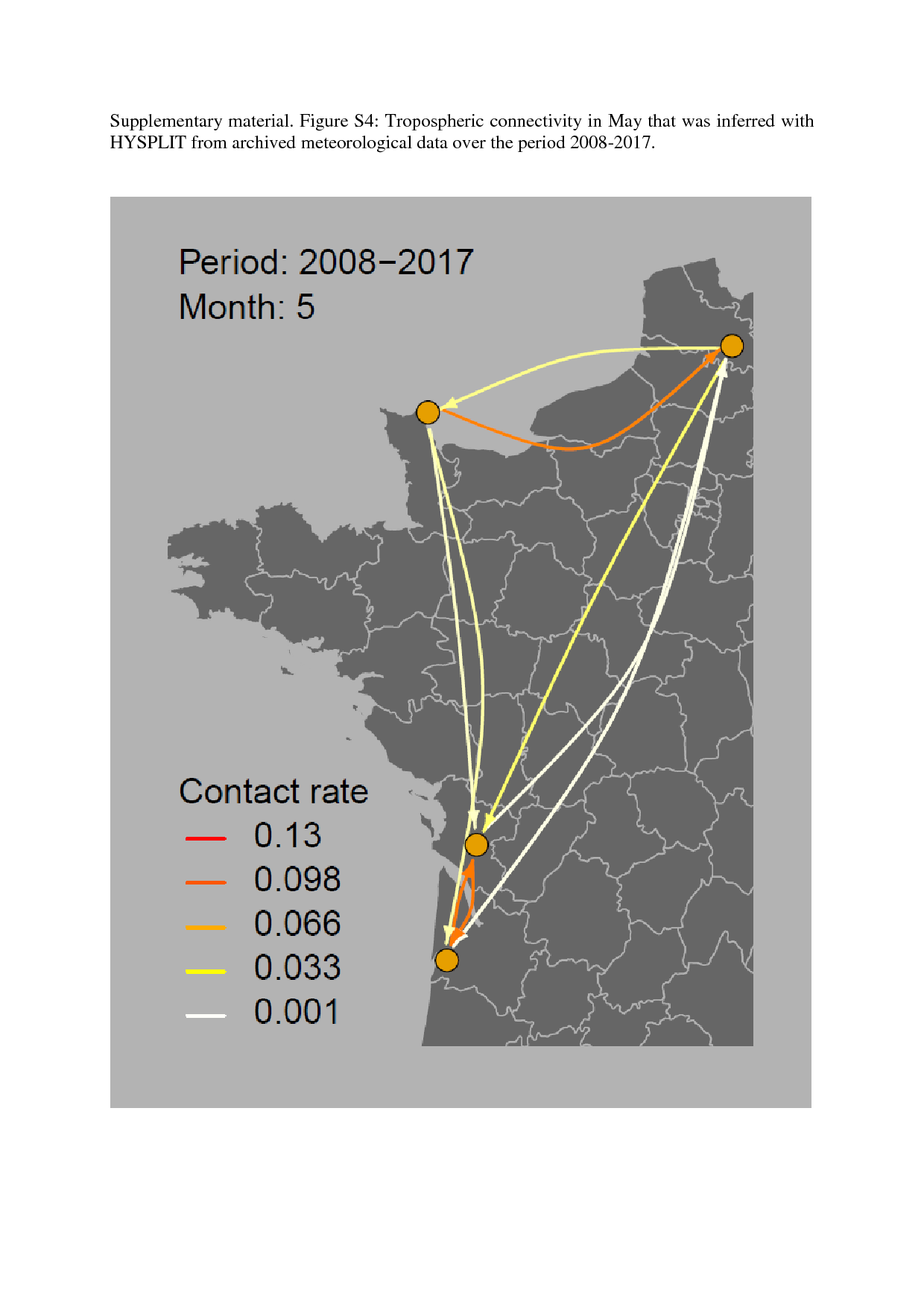

Supplement: Supplementary file 4 [file Image_4.TIFF]

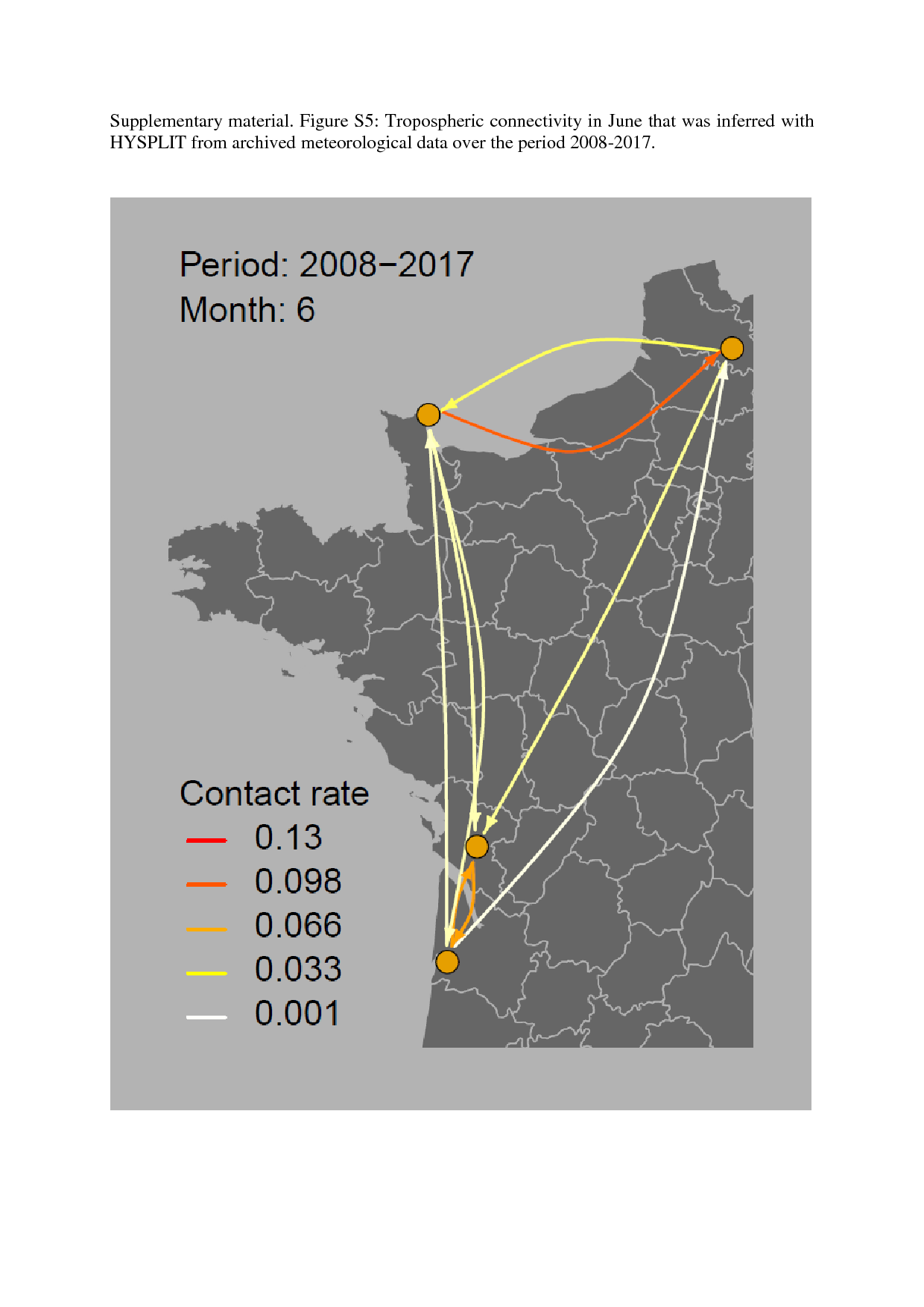

Supplement: Supplementary file 5 [file Image_5.TIFF]

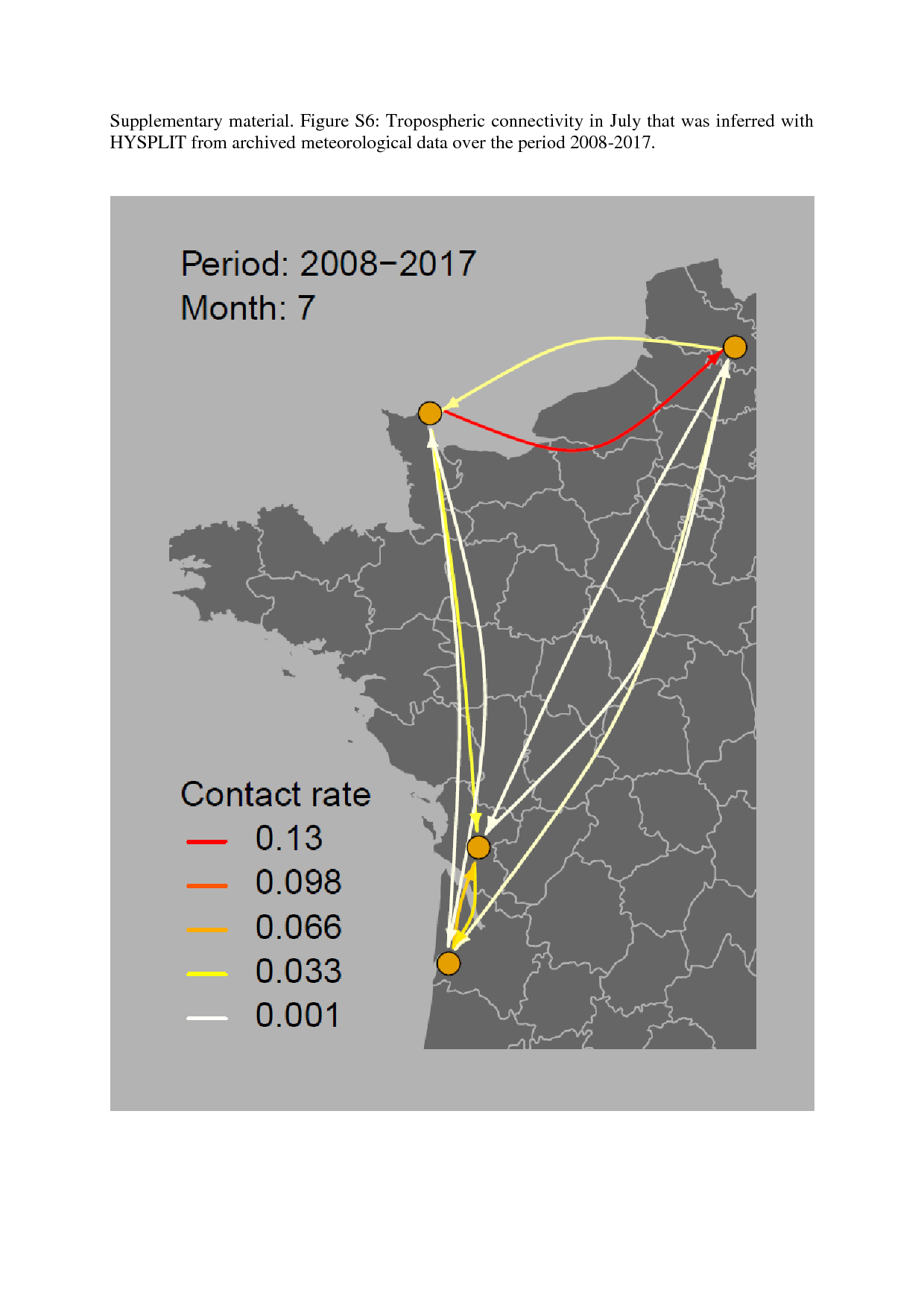

Supplement: Supplementary file 6 [file Image_6.TIFF]

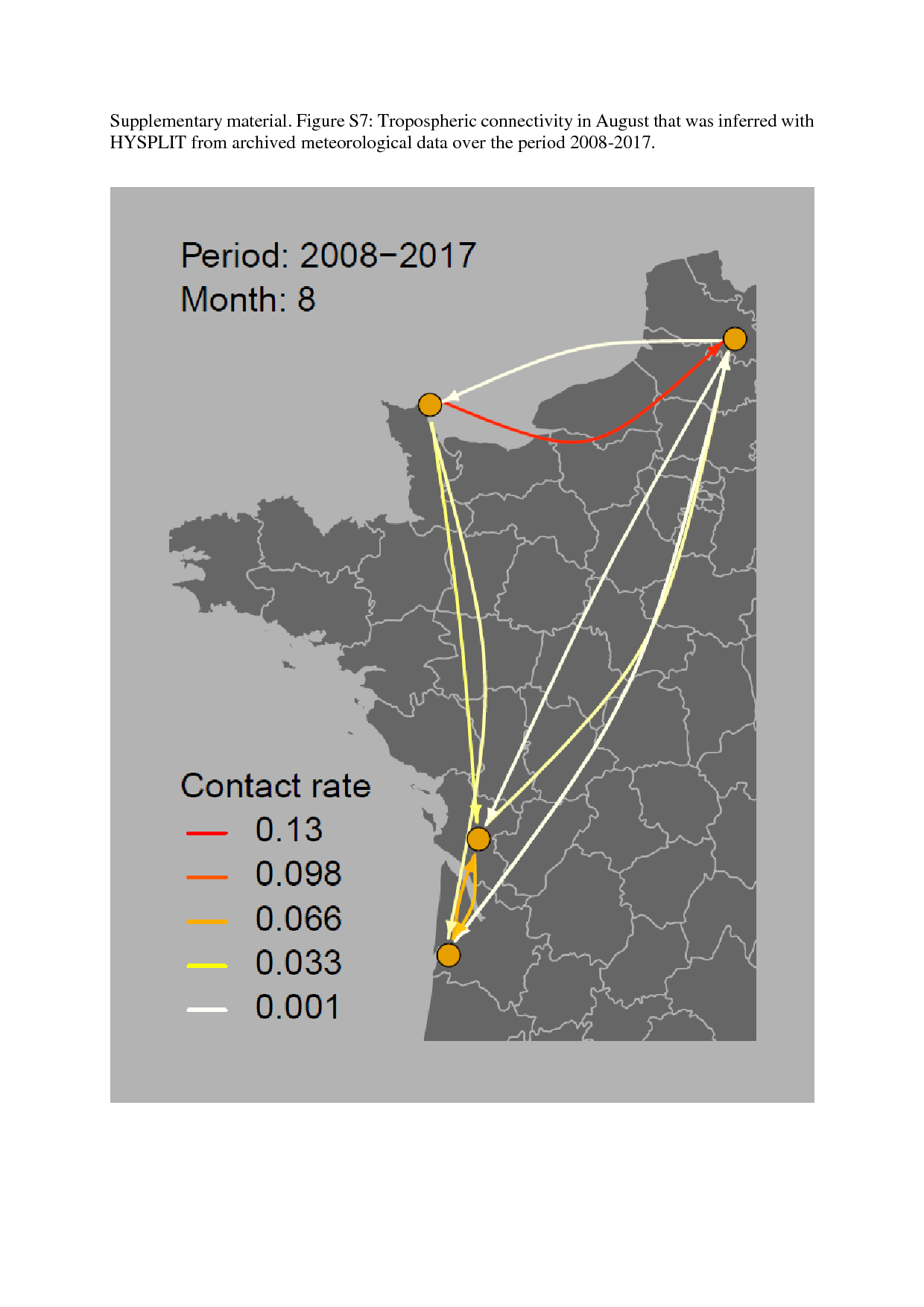

Supplement: Supplementary file 7 [file Image_7.TIFF]

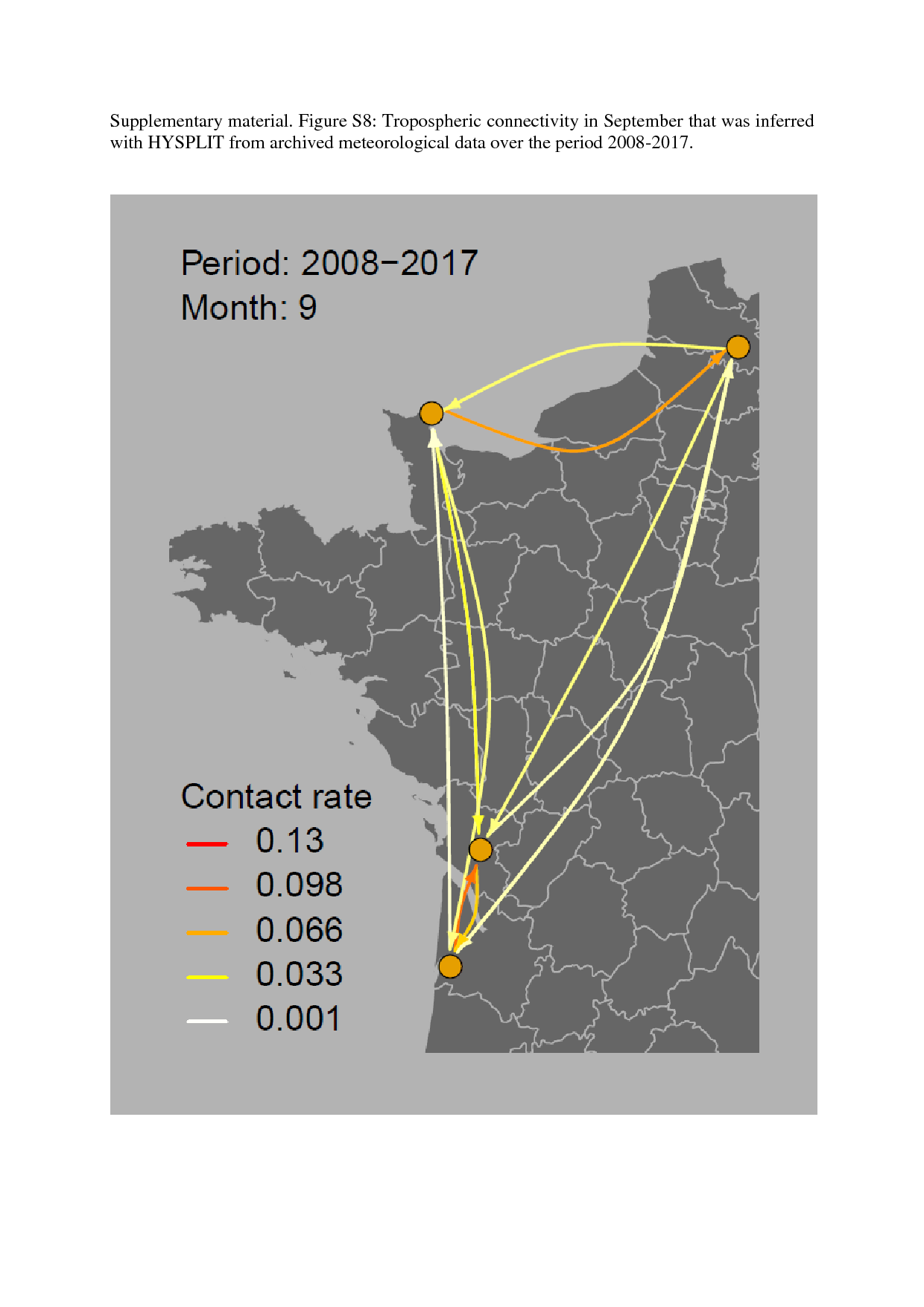

Supplement: Supplementary file 8 [file Image_8.TIFF]

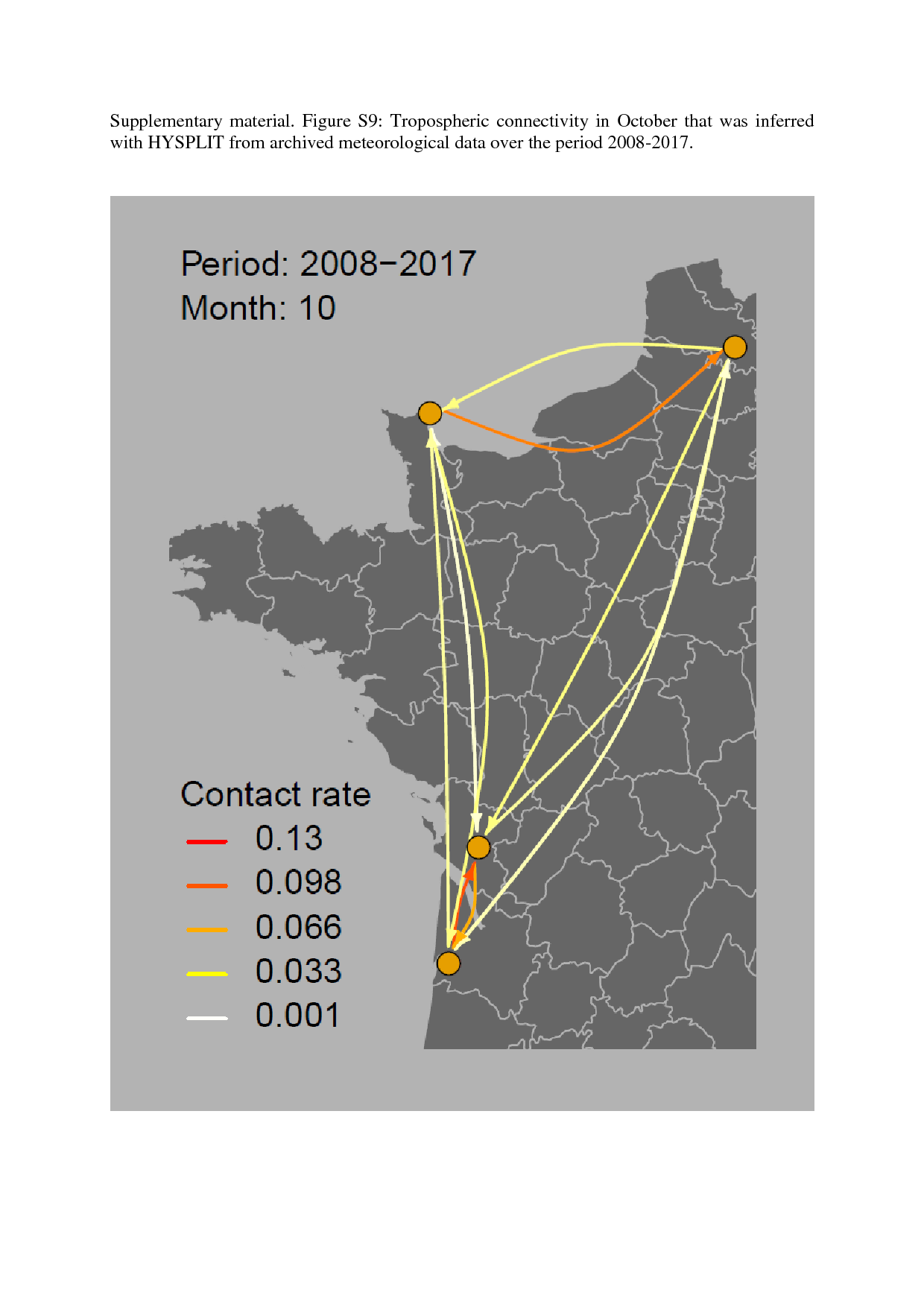

Supplement: Supplementary file 9 [file Image_9.TIFF]

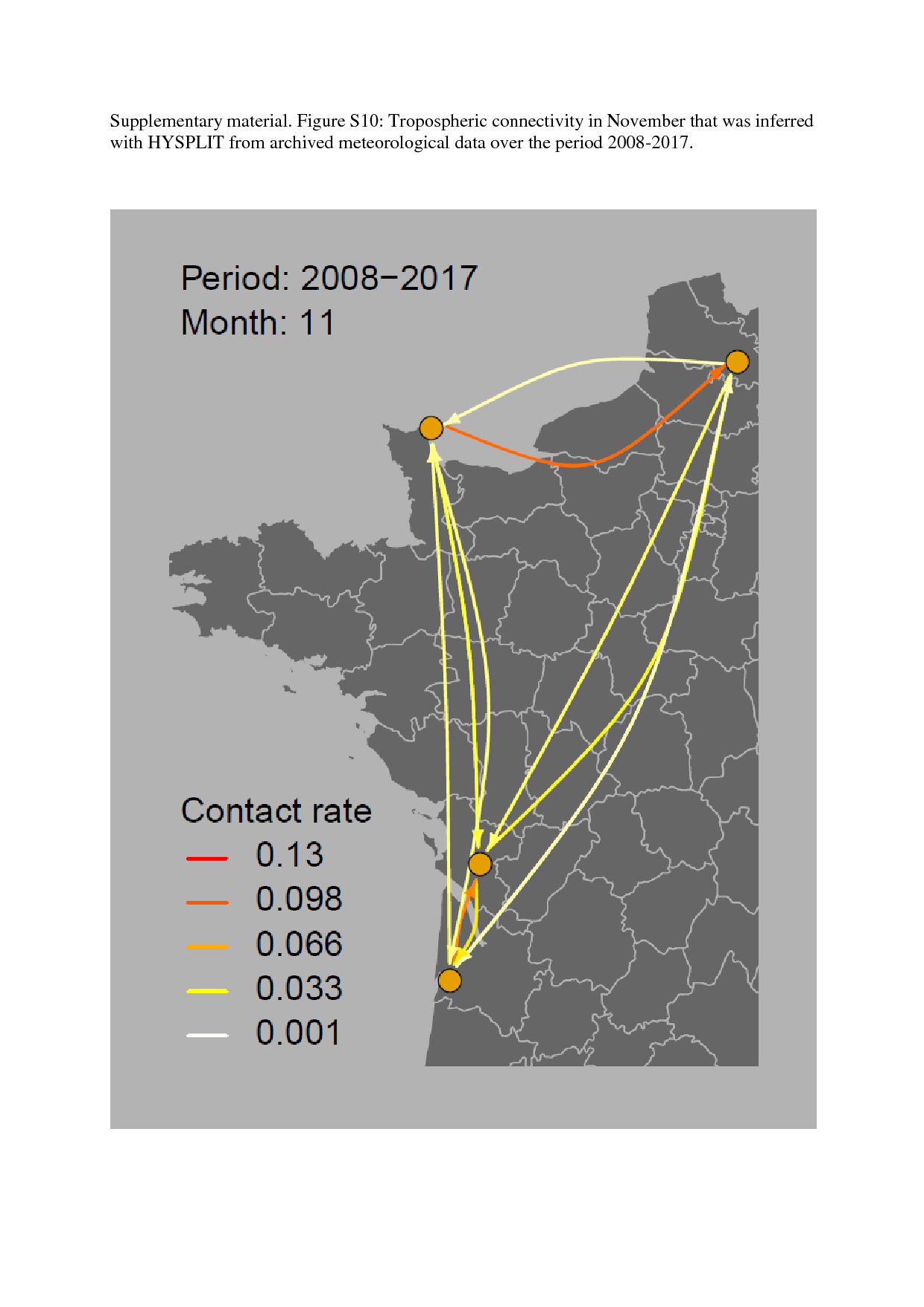

Supplement: Supplementary file 10 [file Image_10.TIFF]

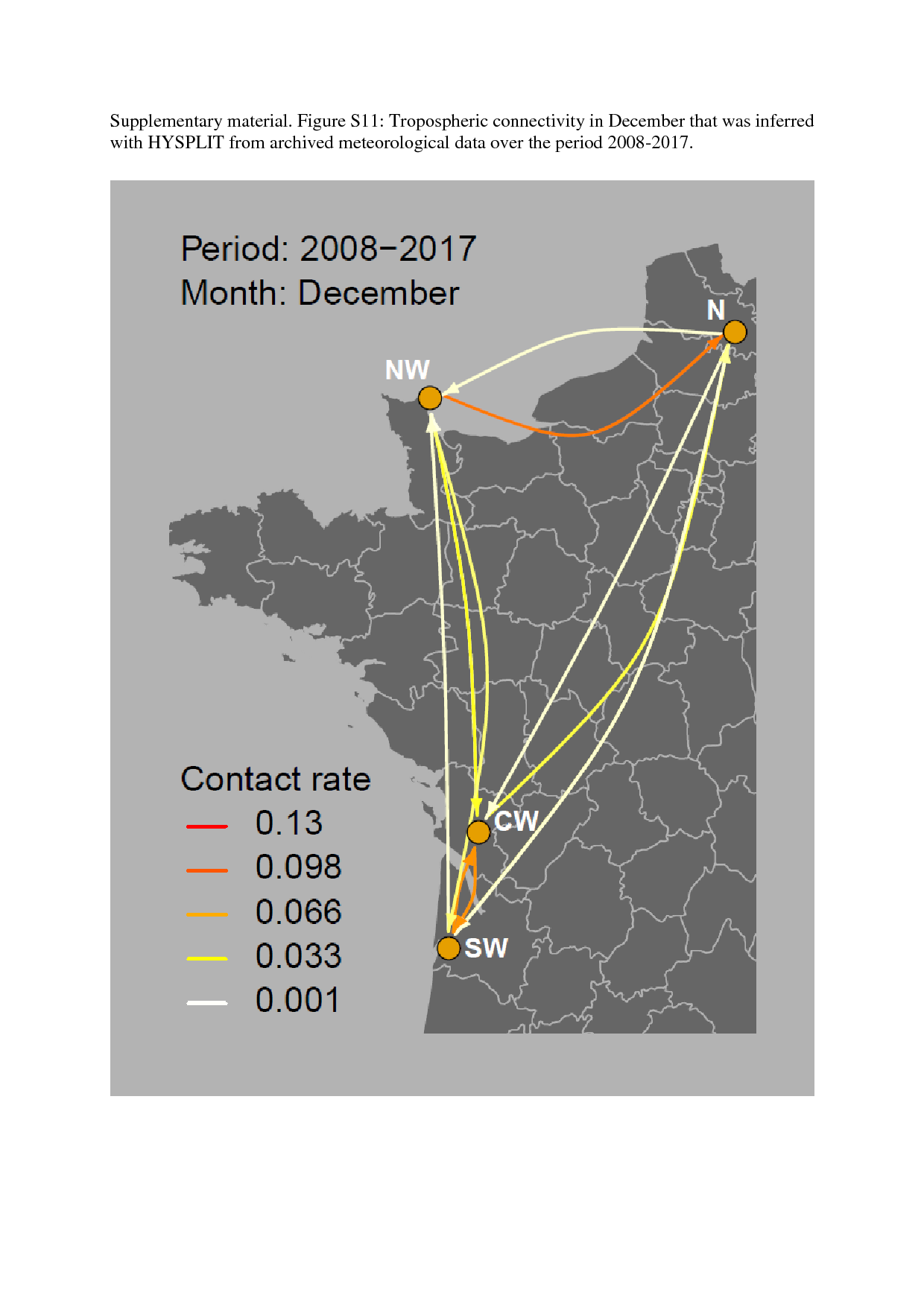

Supplement: Supplementary file 11 [file Image_11.TIFF]
